# Idiotypic DNA vaccination for the treatment of multiple myeloma: safety and immunogenicity in a phase I clinical study

**DOI:** 10.1007/s00262-015-1703-7

**Published:** 2015-05-16

**Authors:** Katy J. McCann, Rosemary Godeseth, Lindsey Chudley, Ann Mander, Gianfranco Di Genova, Paul Lloyd-Evans, Jonathan P. Kerr, Vladimir B. Malykh, Matthew W. Jenner, Kim H. Orchard, Freda K. Stevenson, Christian H. Ottensmeier

**Affiliations:** Experimental Cancer Medicine Centre Southampton and Cancer Sciences Unit, Faculty of Medicine, University of Southampton, Somers Cancer Research Building, Mailpoint 824, Southampton General Hospital, Tremona Road, Southampton, SO16 6YD UK; University Hospital Southampton NHS Foundation Trust, Southampton General Hospital, Tremona Road, Southampton, SO16 6YD UK; NHS Blood and Transplant, Clinical Biotechnology Centre, University of Bristol, Langford House, Lower Langford, Bristol, BS40 5DU UK; University of Exeter Medical School, Royal Devon and Exeter NHS Foundation Trust, Barrack Road, Exeter, EX2 5DW UK

**Keywords:** Multiple myeloma, Immunotherapy, Cancer vaccine, DNA vaccine, Idiotype

## Abstract

We report on the safety and immunogenicity of idiotypic DNA vaccination in a phase I, non-randomised, open-label study in patients with multiple myeloma. The study used DNA fusion gene vaccines encoding patient-specific single chain variable fragment, or idiotype (Id), linked to fragment C (FrC) of tetanus toxin. Patients in complete or partial response following high-dose chemotherapy and autologous stem cell transplant were vaccinated intramuscularly with 1 mg DNA on six occasions, beginning at least 6 months post-transplant; follow-up was to week 52. Fourteen patients were enrolled on study and completed vaccinations. Idiotypic DNA vaccines were well tolerated with vaccine-related adverse events limited to low-grade constitutional symptoms. FrC- and Id-specific T-cell responses were detected by ex vivo ELISPOT in 9/14 and 3/14 patients, respectively. A boost of pre-existing anti-FrC antibody (Ab) was detected by ELISA in 8/14 patients, whilst anti-Id Ab was generated in 1/13 patients. Overall, four patients (29 %) made an immune response to FrC and Id, with six patients (43 %) responding to FrC alone. Over the 52-week study period, serum paraprotein was undetectable, decreased or remained stable for ten patients (71 %), whilst ongoing CR/PR was maintained for 11 patients (79 %). The median time to progression was 38.0 months for 13/14 patients. Overall survival was 64 % after a median follow-up of 85.6 months.

**Electronic supplementary material:**

The online version of this article (doi:10.1007/s00262-015-1703-7) contains supplementary material, which is available to authorized users.

## Introduction

Multiple myeloma (MM) is a B-cell neoplasia characterised by the clonal proliferation of malignant plasma cells in the bone marrow (BM). Despite improved outcomes through novel treatments and autologous stem cell transplantation (ASCT) combined with high-dose chemotherapy (HDT), for most patients the disease remains incurable (www.cancer.org/cancer/multiplemyeloma/) [1, 2]. Current treatments are aimed at providing deep clinical responses alongside supportive care [3, 4]. The need for alternative therapeutic approaches is clear, in particular those that target and eliminate minimal residual disease following ASCT, a scenario in which immunotherapy may prove valuable.

Malignant plasma cells secrete a monoclonal immunoglobulin (Ig), paraprotein, which can be detected in the serum and/or urine of patients. The monoclonal Ig expresses tumour-specific antigenic determinants called idiotopes, collectively termed idiotype (Id). Id are formed by the rearrangement of *variable* (*V*) region genes of the Ig heavy (*V*_*H*_) and light (*V*_*L*_) chains during B-cell maturation within the BM and subsequent somatic hypermutation within a germinal centre reaction following antigen encounter. Tumour-derived Id represents a tumour-specific antigen distinguishable from normal cells or plasma cells and, therefore, provides a unique target for immunotherapy [5, 6].

Prophylactic vaccination with tumour-derived Id has been shown to protect against subsequent tumour challenge in murine models of B-cell lymphoma and myeloma [7, 8]. Such data have led to clinical testing of idiotypic vaccines for the treatment of B-cell malignancies [9–11]; the most recent phase III study in follicular lymphoma demonstrated an increase in disease-free survival following vaccination with hybridoma-derived Id [12]. However, in contrast to lymphoma where the Id is membrane bound, in MM Id is secreted, with little cell-surface expression [13]. Therefore, effective idiotypic vaccination in MM will require the induction of Id-specific T cells that are capable of recognising Id-derived peptides presented on MHC class I and class II molecules. Wen et al. [14] demonstrated that autologous Id-specific cytotoxic T cells of both CD8^+^ and CD4^+^ lineage generated from MM patients can lyse Id-pulsed autologous dendritic cells and autologous myeloma plasma cells, providing proof of concept, with the existence of cytotoxic CD4^+^ T cells increasingly recognised in recent decades [15]. Idiotypic vaccination in MM has been examined in clinical trials, where both humoral and cellular immunological responses have been reported; however, clinical responses have been infrequent [10, 16–18].

Since Id is a self-protein, inducing sufficient immunogenicity is one challenge of vaccination. Our strategy uses a DNA fusion vaccine design to overcome the limited immunogenicity of Id [19]. Patient-specific Id is firstly assembled as a single chain variable fragment (scFv) encoding the tumour-derived *V*_*H*_ and *V*_*L*_ region genes and next fused to *fragment C* (*FrC*) of tetanus toxin, an immune alert signal shown to significantly enhance the immunological response [7, 20, 21]. This DNA fusion vaccine design has distinct advantages as an immunotherapeutic strategy, including the ease of manufacture and administration, the engagement of diverse immune mechanisms to attack tumour cells, including the innate system, and the capacity to overcome potential tolerance to the tumour [7, 19, 22].

Here, we present a phase I clinical trial using idiotypic DNA fusion vaccination in patients with MM and examine the safety and efficacy of this approach.

## Materials and methods

The following materials and methods section is compliant with minimal information about T-cell assay (MIATA) reporting (www.miataproject.org) [23]; further details are provided in Supplementary MIATA Information.

### Patient cohort

Patients with newly diagnosed MM fulfilling WHO criteria, a performance status of ≤1 and who had received ASCT/HDT in the first response were eligible [24]; patients with light chain only or non-secretory disease were excluded. The study was conducted in compliance with ICH-GCP and informed consent was provided by all participants following review and approval by the Medicines and Healthcare Regulatory Authority, the Gene Therapy Advisory Committee and the Local Research Ethics Committee.

### Study design

The study was a phase I, non-randomised, open-label study of DNA vaccination without dose escalation. Patients were vaccinated ≥6 months post-ASCT/HDT if a complete or partial response (CR/PR) or stable disease (SD) was achieved [25]. One milligram of patient-specific *scFv*-*FrC* DNA fusion vaccine was injected intramuscularly on 6 occasions (week 0, 1, 2, 4, 8 and 12). On-study follow-up was at weeks 0, 1, 2, 4, 8 and 12 following vaccination, monthly to week 32 and 3 monthly to week 52. Peripheral blood samples were collected for the evaluation of vaccine immunogenicity. Full blood count, serum biochemistry, paraprotein and beta-2 microglobulin analyses were performed by the Department of Immunology, University Hospital Southampton NHS Foundation Trust. Time to progression (TTP) and overall survival (OS) were recorded from the date of ASCT.

### Patient material

An anti-coagulated BM aspirate was received fresh at diagnosis; mononuclear cells were separated by centrifugation over lymphoprep™ (Axis-Shield PoC AS, Oslo, Norway) according to the manufacturer’s instructions. Viable cells were stored in liquid nitrogen in aliquots of 5–10 × 10^6^ cells/mL of freezing medium (10 % dimethylsulphoxide, 50 % decomplemented human AB serum and 40 % RPMI) until *V* gene identification. Pre-treatment serum (>10 mL) was harvested from clotted whole blood by centrifugation and stored in aliquots of 5 mL at −80 °C until paraprotein purification. Peripheral blood mononuclear cells (PBMCs) were isolated from on-study blood collections by centrifugation over lymphoprep™ (Axis-Shield PoC AS) as described above; 5–10 × 10^6^ viable cells/mL freezing medium were stored in liquid nitrogen. On-study serum was harvested by centrifugation and stored in aliquots of 1 mL at −80 °C.

### Construction of patient-specific *scFv-FrC* DNA fusion vaccines

Procedures relating to the identification of tumour-derived *V* genes used in this study have been published previously [26]; total RNA was extracted from 5 to 10 × 10^6^ tumour cells, followed by cDNA synthesis and PCR amplification for *V*_*H*_ and *V*_*L*_ genes using standard primer combinations and cycling conditions [20]. Tumour-related *V* genes were defined by the presence of repeated sequences with a clonally related complementarity determining region 3; sequence alignment analysis used MacVector software (Oxford Molecular, Oxford, UK) and aligned to the IMGT database (www.imgt.org). Tumour-derived *V*_*H*_ and *V*_*L*_ gene sequences were assembled as scFv, linked at the C-terminus to *FrC* and cloned into pcDNA3 vector (Invitrogen Limited, Paisley, UK) as previously described [7, 20]; vaccine design is shown in Supplementary Fig 1. Patient-specific vaccines were produced to GMP standard at NHS Blood and Transplant, Clinical Biotechnology Centre, University of Bristol, and stored in sterile PBS at −80 °C until clinical use.

### Generation of patient-specific Id and FrC proteins for immunological endpoint evaluation

Assembly and expression of recombinant FrC and patient-specific scFv proteins were as previously described [27]; FrC and scFv proteins were tagged at the C-terminus with kappa chain constant region and expressed using the mammalian FreeStyle™293 expression system (Invitrogen Ltd.) according to the manufacturer’s instructions. Purification of recombinant proteins used CaptureSelect^®^ Fab kappa affinity matrix (BAC B.V., Naarden, The Netherlands) according to the manufacturer’s instructions. Tumour-derived scFv expression was successful in 11/14 patients; scFv protein was not available for immunomonitoring of patients MM08, MM10 and MM11.

Purification of paraprotein from patient serum used CaptureSelect^®^ human IgG affinity matrix and CaptureSelect^®^ human IgA affinity matrix (BAC B.V.), for IgG (*n* = 8) and IgA (*n* = 5), respectively, according to the manufacturer’s instructions. Purification was not performed for IgD paraprotein (*n* = 1); paraprotein was not available for immunomonitoring of patient MM02.

Final protein concentration was determined by BCA™ protein assay (Perbio Science UK Ltd., Cramlington, UK). Size and purity was confirmed by separation by SDS-PAGE using NuPAGE bis–tris gradient polyacrylamide (4–12 %) gel (Invitrogen Ltd.) followed by staining with SimplyBlue Safestain™ (Invitrogen Ltd.); western blot analysis using polyclonal goat anti-human kappa light chain HRP-conjugated antibody (Ab) (Sigma-Aldrich Company Ltd.) was performed for recombinant scFv and FrC. Specificity of scFv was assessed by ELISA using patient-specific anti-Id Ab generated in C57BL/6 mice (*n* = 8–10) following vaccination with *scFv*-*FrC* DNA vaccine, as previously described [7]; we previously showed anti-sera generated in this way is able to bind idiotypic Ig on the surface of autologous lymphoma cells, as measured by FACS analysis (unpublished observation). Specificity ELISA used polyclonal sheep anti-mouse IgG Ab (The Binding Site, Birmingham, UK) to detect the binding of patient-specific or non-specific control anti-sera to each protein. Endotoxin levels were assessed using the endpoint chromogenic (LAL) kit (Charles River Laboratories International, Inc., Wilmington, MA, USA) according to the manufacturer’s instructions.

### Immunological evaluation

Ab responses to FrC were measured using a validated ELISA and quantified in relative Ab units against a tetanus antitoxin human Ig reference standard (National Institute of Biological Standards and Control, UK), as previously described [28]. For the detection of anti-Id Ab, a 96-well Maxisorp immunoplate (Nunc, Roskilde, Denmark) was coated with 10 µg/mL patient-specific recombinant scFv protein or patient-purified IgA paraprotein; an irrelevant scFv/IgA paraprotein served as a negative control. Patient sera were tested at multiple time-points at a 1:10 dilution, with patient-specific mouse anti-sera used as a positive control (1:10). Specific anti-Id Ab was determined at each time-point by subtracting the mean absorbance of the irrelevant control protein from the test protein. All data are expressed as fold increase compared with pre-vaccination baseline (week 0). An antigen-specific response to the vaccine was defined as greater than or equal to twofold increase over pre-vaccination baseline at multiple time-points [++] or a single time-point [+].

Cellular responses to the vaccine were measured on cryopreserved PBMCs by ex vivo IFN-γ (IL-13/IL-2) ELISPOT assay, as described previously [28]. PBMCs (4 × 10^5^ cells/well) were incubated with recombinant FrC (20 µg/mL), recombinant scFv (100 µg/mL) and patient-purified paraprotein (100 µg/mL) for 40 h at 37 °C in 5 % CO_2_; control wells included an irrelevant scFv, an irrelevant isotype-matched paraprotein, medium only and phytohemagglutinin (PHA; 5 µg/mL; Sigma-Aldrich Company Ltd.). Spot-forming cells (SFC)/well were counted using the AID ELISpot Plate Reader System ELR04 and software (AutoImmun Diagnostika GmbH, Strassberg, Germany). Each well was first expressed as SFC/million PBMCs, followed by subtracting the mean spot number of the triplicate of unstimulated cells from the test triplicate [28, 29]; Id-specific responses were obtained by deducting spot values for irrelevant control proteins. A specific response to a test antigen at any given time-point was calculated as mean SFC/million minus mean SFC/million of pre-vaccination baseline and defined as ≥20 SFC/well and ≥2 SDEV above medium-only wells at multiple time-points [++] or a single time-point [+].

### Clinical monitoring

TTP and OS were recorded to event or censor date for all patients on study; data were frozen in September 2013 for analysis. TTP was defined as the time from the date of ASCT to progression of paraprotein, according to the International Myeloma Working Group criteria [30]. OS was defined as the time from the date of ASCT to death.

### Statistical analyses

Statistical analyses were performed with GraphPad Prism software, version 6.0a (GraphPad Software, Inc., La Jolla, USA). A significant antigen-specific response was determined by a Student’s t test with a confidence level of 95 % (*P* < 0.05). The distributions of time to event data were estimated using the Kaplan–Meier method and compared using the log-rank test statistic (Mantel–Cox).

## Results

### Patient characteristics

Following successful vaccine construction, 15 eligible patients were enrolled and received vaccination; one patient was removed from study after disease progression at week 4 and is not included in safety and immunological analyses. Patient MM19 exhibited slowly rising paraprotein at the week 0 visit (vaccination 1), but was otherwise in good general health with no clinical symptoms, and therefore the decision was taken to commence vaccination; symptomatic progression followed at week 43 and the patient was removed from study. Two further patients progressed post-vaccinations and went off study at week 16 (MM04) and week 41 (MM10). The characteristics of 14 patients evaluable for vaccine safety and immunogenicity are shown in Table 1: ten patients were male; mean age was 58 years (range 36–70 years); 13 patients presented with International Staging System stage I or II disease [30]; Ig isotypes were IgG (*n* = 8), IgA (*n* = 5) and IgD (*n* = 1); CR, PR and SD were achieved in seven, six and one patients post-ASCT/HDT, respectively; and mean time from ASCT to first vaccination was 12.5 months (range 6.4–21.4 months).

**Table 1:** Patient characteristics

Patient	Sex/age	Id isotype	Disease stage^a^	Prior therapies	Disease status^b^	Time from ASCT to vaccination (months)
MM01	M/64	IgG/K	II	RT, 4xVAD	PR	9.9
MM02	M/70	IgD/K	I	5xC-VAD	CR	7.6
MM03	M/66	IgG/K	II	6xC-VAD	PR	13.6
MM04	M/69	IgG/L	I	4xCTD	PR	16.5
MM05	F/65	IgG/K	II	4xCTD	PR	6.5
MM07	F/42	IgG/L	II	6xCTD	SD	9.4
MM08	M/64	IgG/K	II	6xVAD	CR	9.9
MM09	M/66	IgG/K	I	6xCTD	PR	9.2
MM10	F/36	IgA/K	II	5xCTD	CR	10.8
MM11	M/51	IgA/K	II	5xCTD	CR	6.4
MM15	F/59	IgA/L	I	5xCTD	CR	19.0
MM17	M/45	IgG/K	I	5xCTD	CR	21.4
MM19	M/62	IgA/L	II	6xC-VAD, 1xM	CR	15.4
MM21	M/54	IgA/K	II/III	6xCTD, 1xM, T	PR	18.7

*CTD* cyclophosphamide/thalidomide/dexamethasone, *C*-*VAD* cyclophosphamide/vincristine/adriamycin/dexamethasone, *M* melphalan, *RT* radiotherapy, *VAD* vincristine/adriamycin/dexamethasone, *T* thalidomide maintenance

^a^Staging of disease used the International Staging System introduced by the International Myeloma Working Group [30]

^b^Status of disease post-ASCT/HDT: complete response (CR), partial response (PR), stable disease (SD)

### Safety and adverse events

The personalised idiotypic DNA fusion vaccines were safe and well tolerated (Table 2). Adverse event (AE) reporting was according to the Common Terminology Criteria for Adverse Events, version 4.03 (evs.nci.nih.gov). Eleven of 14 patients (79 %) reported AEs whilst on study. Five grade 3 AEs were reported, but were assessed as vaccine unrelated: pulmonary infection, chest pain, deep vein thrombosis, maculo-papular rash due to an allergic reaction and hospitalisation with head injury following a car accident. Vaccine-related AEs were grade 1 or 2 and consisted of constitutional symptoms, including flu-like symptoms (5/14 patients, 7 events), fatigue (4/14 patients, 7 events), musculoskeletal aches (5/14 patients, 7 events) and skin injection site reactions (2/14, 2 events).

**Table 2:** Adverse events

Adverse event^a^	Grade 1	Grade 2	Grade 3
Cardiac disorders			
Chest pain			1 (1.6 %)
Tachycardia	1 (1.6 %)		
Gastrointestinal			
Abdominal pain	2 (3.2 %)		
Diarrhoea	1 (1.6 %)		
Dyspepsia	1 (1.6 %)		
Nausea	1 (1.6 %)		
Periodontal disease—gingivitis	1 (1.6 %)		
Vomiting	2 (3.2 %)		
General disorders and administration site reactions			
Oedema—limb	1 (1.2 %)		
Fatigue	7 (11.3 %)		
Fever	1 (1.6 %)	1 (1.6 %)	
Flu-like symptoms	6 (9.7 %)	1 (1.6 %)	
Injection site redness, rash	2 (3.2 %)		
Other, diaphoresis	1 (1.6 %)		
Infections and infestations			
Eye	1 (1.6 %)		
Lung		1 (1.6 %)	
Upper respiratory		3 (4.8 %)	1 (1.6 %)
Other, varicella zoster virus	1 (1.6 %)		
Metabolism and nutrition disorder			
Anorexia	1 (1.6 %)		
Musculoskeletal and connective tissue disorders			
Arthralgia	1 (1.6 %)		
Arthritis	1 (1.6 %)		
Back pain	1 (1.6 %)		
Chest wall pain	1 (1.6 %)		
Myalgia	2 (3.2 %)		
Shoulder pain	1 (1.6 %)		
Nervous system disorders			
Lethargy	1 (1.6 %)		
Neuralgia	1 (1.6 %)		
Psychiatric disorders			
Confusion	1 (1.6 %)		
Depression	1 (1.6 %)		
Respiratory, thoracic and mediastinal disorders			
Cough	3 (4.8 %)		
Pharyngolaryngeal pain	3 (4.8 %)		
Pleuritic pain	1 (1.6 %)		
Skin and subcutaneous tissue disorders			
Pruritus	1 (1.6 %)		
Rash maculo-papular	1 (1.6 %)		1 (1.6 %)
Social circumstances			
Social circumstances—other			1 (1.6 %)
Vascular disorders			
Hypertension		1 (1.6 %)	
Thromboembolic event			1 (1.6 %)

^a^Recording of adverse events used the Common Terminology Criteria for Adverse Events, version 4.03

### Immunological evaluation

Serum and PBMCs were collected from each patient at defined time-points on study for subsequent immunological analysis; immune responses were assessed by ELISA and ex vivo ELISPOT assay and are summarised in Table 3.

**Table 3:** Summary of immune responses and clinical outcome

Patient	Immune responses^a^				Clinical outcome	
	FrC-specific		Id-specific^b^		TTP^c^ (months)	OS^d^ (months)
	Humoral	Cellular	Humoral	Cellular		
MM01	++	++	−^S^	+^P^	32.9	45.8
MM02	−	−	−^S^	−^S^	56.0	96.1^+^
MM03	+	++	−^S^	−^S,P^	48.0	98.8
MM04	++	+	−^S^	−^S,P^	19.5	49.4
MM05	++	++	−^S^	++^S^, −^P^	50.5	106.5^+^
MM07	−	+	−^S^	−^S^, +^P^	21.0	92.1^+^
MM08	−	−	na	−^P^	64.4	105.5^+^
MM09	−	−	−^S^	−^S,P^	38.0	102.0^+^
MM10	−	+	−^P^	−^P^	14.7	37.7
MM11	++	+	−^P^	−^P^	46.0	79.0^+^
MM15	−	−	−^S,P^	−^S,P^	95.1^+^	95.1^+^
MM17	++	+	−^S^	−^S,P^	35.0	65.1^+^
MM19	++	−	++^S^, −^P^	−^S,P^	15.4	34.7
MM21	++	++	−^S^	−^S,P^	45.0	45.0^+^
Total immune responses	8/14	9/14	1/13	3/14		
	10/14		4/14			

*na* not assayed

^a^Positive immune response criteria: for ELISA, greater than or equal to twofold increase over pre-vaccination baseline at multiple time-points (++) or a single time-point (+); for ELISPOT, mean SFC minus baseline SFC of ≥20 SFC/well and ≥2 SD above medium-only wells at multiple time-points (++) or a single time-point (+)

^b^Immune responses were assayed using patient-purified paraprotein (^P^) and/or patient-derived recombinant scFv (^S^)

^c^TTP was defined as the time from the date of ASCT to progression of paraprotein, according to the International Myeloma Working Group criteria [30] or to the point of data censor (September 2013): ^+^CR maintained

^d^OS was defined as the time from the date of ASCT to death or to the point of data censor (September. 2013): ^+^alive

**Fig. 1:**
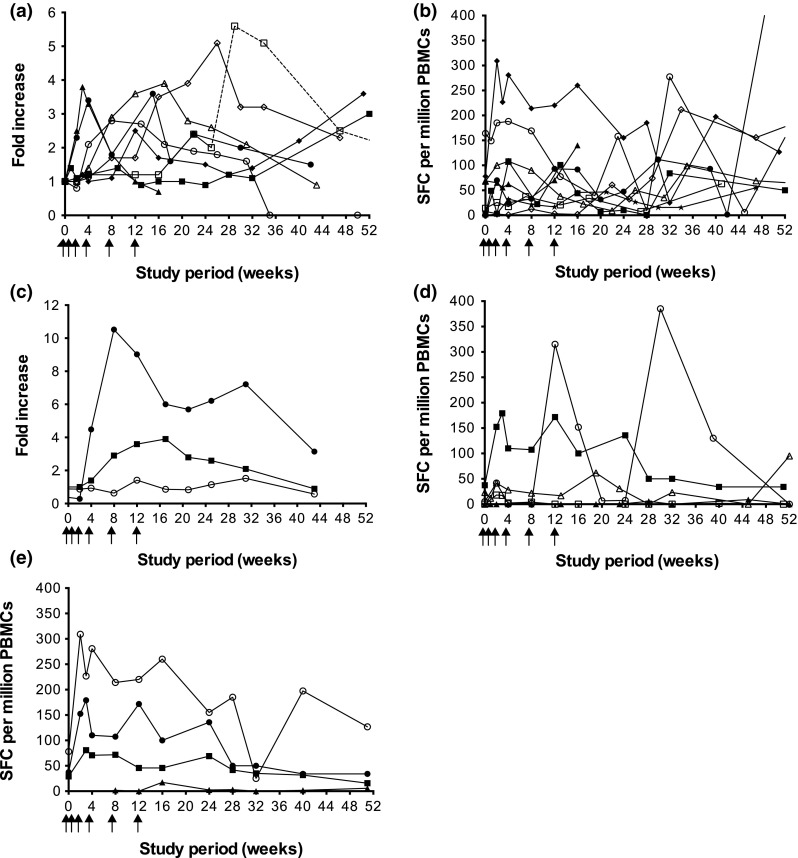
Vaccine-induced immune responses. Following vaccination with *scFv*-*FrC* DNA fusion vaccine, humoral and cellular immune responses to both FrC and Id were monitored in the blood of patients at regular time-points over the 52-week study period; detection was by ELISA and ex vivo IFN-γ ELISPOT, respectively, and the criteria for positive responses are as described in the “Materials and methods” section and Supplementary MIATA Information. **a** Eight of 14 patients generated a boost in anti-FrC IgG Ab following vaccination; MM01 (c*losed circles*), MM03 (*closed squares*), MM04 (*closed triangles*), MM05 (*closed diamonds*), MM11 (*open circles*), MM17 (*open squares*), MM19 (*open triangles*) and MM21 (*open diamonds*). Data are expressed as fold increase over pre-vaccination baseline. Patient MM17 received a tetanus booster vaccination at week 25 on study as part of routine vaccinations for travel purposes, as indicated by a *dashed line*. **b** Nine of 14 patients developed a FrC-specific T-cell response following vaccination; MM01 (c*losed circles*), MM03 (*closed squares*), MM04 (*closed triangles*), MM05 (*closed diamonds*), MM07 (*open circles*), MM10 (*open squares*), MM11 (*open triangles*), MM17 (*open diamonds*) and MM21 (*closed stars*). **c** One of 13 patients (MM19) generated an anti-Id IgG Ab response following vaccination, detectable against scFv (*closed circles*), but not paraprotein (*open circles*). An irrelevant scFv target derived from patient MM05 was used to establish the specificity of the response. The FrC-specific IgG Ab response for this patient is shown for comparison (*closed squares*). Data are expressed as fold increase over pre-vaccination baseline. **d** Three of 14 patients developed an Id-specific T-cell response following vaccination, which was directed against either scFv (*n* = 1; *closed symbols*) or Ig paraprotein (*n* = 2; *open symbols*); MM01 (*circles*), MM05 (*squares*) and MM07 (*triangles*). **e** Id-specific T cells generated following vaccination in patient MM05 secreted IFN-γ (*closed circles*) and IL-13 (*closed squares*), but not IL-2 (*closed triangles*), upon stimulation with scFv; stimulation with an irrelevant scFv target derived from patient MM09 was used to establish specificity of the response. The FrC-specific T-cell response (IFN-γ-release) for this patient is shown for comparison (*open circles*)

Ten of 14 patients (71 %) developed an immune response to FrC following vaccination (Table 3). Eight patients (57 %) boosted anti-FrC IgG Ab, of which seven exhibited a strong response [++]. Ab response kinetics are shown in Fig. 1a; the maximum response was 3.6-fold (week 15), 3.0-fold (week 52), 3.8-fold (week 3), 3.6-fold (week 51), 2.8-fold (week 8), 5.6-fold (week 29), 3.9-fold (week 17) and 5.1-fold (week 26) for MM01, MM03, MM04, MM05, MM11, MM17, MM19 and MM21, respectively. A FrC-specific T-cell response was observed in 9 patients (64 %), of which four displayed a strong response [++] (Table 3; Fig. 1b).

Immune responses to Id were assessed against patient-derived recombinant scFv protein and/or patient-purified paraprotein. We found no evidence for pre-existing Id-specific cellular or humoral immune responses in any of the patients. Overall, 4/14 patients (29 %) developed an immune response to Id (Table 3). Anti-Id IgG ELISA was performed for 13/14 patients against either recombinant scFv protein (*n* = 9), IgA paraprotein (*n* = 2) or both (*n* = 2). Following vaccination, 1/13 patients (8 %; MM19) generated an anti-Id IgG Ab response [++] detectable with recombinant scFv, the kinetics of which paralleled that to FrC; the maximum anti-Id response was 10.5-fold (week 8), and anti-Id Ab was maintained for the length of the study (Fig. 1c). No anti-Id IgG Ab response was observed when assayed with patient-purified IgA paraprotein. Anti-Id ELISPOT was performed for all patients against either recombinant scFv protein (*n* = 1), Ig paraprotein (*n* = 4) or both (*n* = 9). Three of 14 patients (21 %) developed an Id-specific T-cell response following vaccination (Table 3; Fig. 1d); in each case, a FrC-specific humoral and/or cellular response was also observed. Two patients (MM01 and MM07) demonstrated anti-Id T cells [+] upon stimulation with patient-purified IgG paraprotein, but not recombinant scFv in the case of MM07; due to limited PBMCs, ELISPOT was not performed against recombinant scFv for patient MM01. Conversely, patient MM05 exhibited a strong T-cell response [++] to recombinant scFv, but not patient-purified IgG paraprotein, with a maximum increase in IFN-γ and IL-13 production of 4.8-fold and 2.8-fold, respectively (week 3); no IL-2 secretion was observed (Fig. 1e). The response to scFv paralleled that to FrC.

### Clinical outcome

**Fig. 2:**
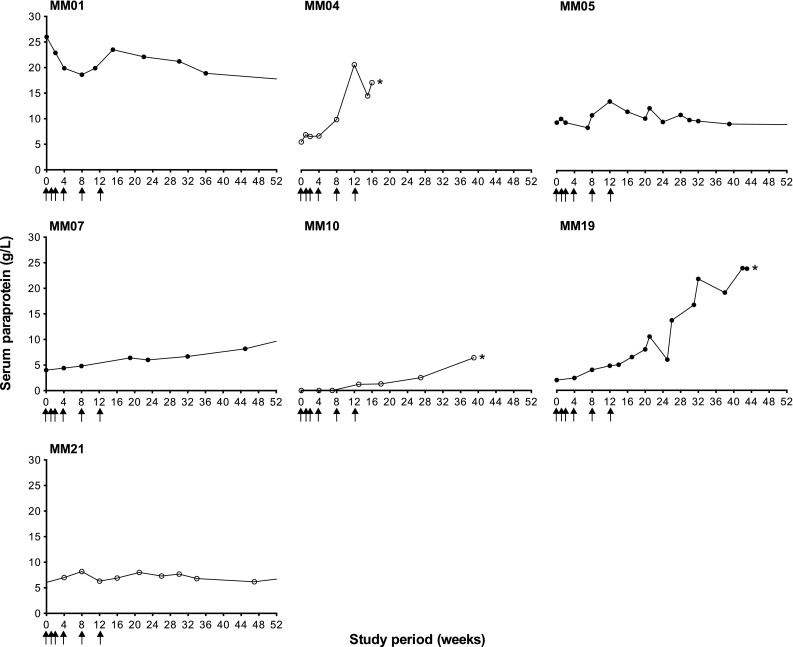
Serum paraprotein. The concentration of serum paraprotein (g/L) was monitored at regular time-points over the 52-week study period for all patients; 7/14 patients displayed detectable paraprotein during the study period. Serum paraprotein decreased or remained stable for one and two patients, respectively. Four patients exhibited rising paraprotein, which was indicative of disease progression in three patients who were subsequently removed from study before the completion of follow-up, as indicated by an *asterisk*: MM04 (week 16), MM10 (week 41) and MM19 (week 43). *Closed circles* represent Id responders (*n* = 4) and *open circles* represent Id non-responders (*n* = 3). Serum protein electrophoresis was performed by the Department of Immunology, University Hospital Southampton NHS Foundation Trust

Serum paraprotein was undetectable in eight patients at the onset of vaccination (week 0) and remained so for the full 52-week study period in seven patients (50 %). The concentration of paraprotein at pre-vaccination baseline ranged from 2.0 to 26.0 g/L (mean 8.8 g/L; *n* = 6). Figure 2 shows the change in paraprotein concentration for all patients in which paraprotein was detectable at any point during the study (*n* = 7), including the four Id responders: MM01, MM05, MM07 and MM19. One patient (7 %; MM01) exhibited a decrease in serum paraprotein by 8.9 g/L, whilst paraprotein remained stable, with some minor fluctuations (<1.0 g/L), in two patients (14 %; MM05 and MM21). In four patients (29 %), serum paraprotein increased (mean 11.4 g/L, range 5.9–21.8 g/L), with three patients removed from study due to disease progression: MM04 at week 16 (IgG paraprotein increase of 11.6 g/L plus clinical progression), MM10 at week 41 (IgA paraprotein increase of 6.4 g/L, asymptomatic) and MM19 at week 43 (IgA paraprotein increase of 21.8 g/L plus clinical progression).

TTP and OS were assessed and recorded for all patients (Table 3; Supplementary Fig 2). By the end of on-study follow-up, 11 patients (79 %) remained in ongoing CR/PR, which decreased to one patient (7 %) by the censor date; post-ASCT/HDT, ongoing CR/PR was maintained for 3+ years in eight patients (57 %), for 4+ years in five patients (36 %) and for 5+ years in two patients (14 %). Median TTP was 38.0 months for 13/14 patients; median TTP was 27.0 months for Id responders (*n* = 4) compared with 45.0 months for Id non-responders (*n* = 9) (Supplementary Fig 2a; log-rank *P* value = 0.206). OS was 64 % (9/14 patients) after a median follow-up of 85.6 months (range 34.7–106.5 months), with all deaths due to end stage MM; there was no significant difference in OS between Id responders and Id non-responders (Supplementary Fig 2b; log-rank *P* value = 0.430).

## Discussion

The present phase I clinical trial employed patient-specific DNA fusion vaccination to target MM; vaccines were composed of tumour-derived scFv linked to FrC of tetanus toxin, which has previously shown to enhance the immune response to Id [7, 20, 21]. Individual *scFv*-*FrC* DNA fusion vaccines were constructed and safely delivered to 14 patients with myeloma following treatment with ASCT/HDT; no vaccine-related AEs of grade 3 or above were observed. Vaccine-induced immune responses were examined: 71 and 29 % of patients generated responses to FrC and Id, respectively. The majority of patients maintained favourable serum paraprotein levels (71 %) and upheld ongoing CR/PR (79 %) during the 52-week study period. Median TTP was 38.0 months for 13 patients, and OS was 64 % after a median follow-up of 85.6 months.

Patients with MM have a decreased ability to mount a response to vaccination due to widespread immunosuppressive treatment regimens [31]. In 29 % of our patients, vaccination failed to provoke a cellular or humoral response to the immune alert signal FrC, suggestive of persistent immunodeficiency since vaccine delivery was not compromised at any time. Vaccination was initiated ≥6 months post-ASCT/HDT. We have previously shown that MM patients demonstrate substantial recovery of responsiveness to tetanus toxin vaccination within 6 months–1 year following treatment [32]. In this period, myeloma burden is predicted to be at its lowest and immune reconstitution to be well advanced [25]. Moreover, low disease burden is known to be associated with augmented responses to anti-tumour vaccination [33]. The recovery of immune competence is evident from a successful expansion of FrC-specific immune responses in 71 %; mean time between ASCT/HDT and vaccination for FrC responders and FrC non-responders was not significantly different at 12.9 and 11.4 months, respectively (*P* value = 0.474).

Overall, our DNA fusion vaccine approach resulted in the generation of Id-specific immune responses in 29 % of patients, detectable in peripheral blood; we believe that this is a clinically relevant response rate as published data show that numbers of Id-specific T cells occur in the blood and BM at a similar frequency [34]. Our vaccine was designed to provide CD4^+^ T-cell help through the FrC component [7], as well as activate the innate immune system through the plasmid backbone. We show that the anti-Id responses achieved are comparable to those generated in MM patients vaccinated against influenza virus, which have been reported to be as low as 19 % [35]. We report vaccine-induced anti-Id Ab for one patient despite a rising paraprotein sufficient to require further treatment and early removal from the study. Since myeloma cells secrete tumour-specific monoclonal Ig, it is traditionally considered that anti-Id Ab is not effective against plasma tumour cells because (1) the large amount of circulating soluble paraprotein may bind and neutralise the anti-Id Ab and (2) the absence of surface Ig on tumour cells renders them resistant to the effect of anti-Id Ab. Yet, Moshitzky et al. [36] demonstrate that anti-Id Ab is capable of inhibiting the growth of myeloma cells in the absence of membrane-bound Ig in the murine D2 plasmacytoma model suggesting anti-Id Ab could confer clinical benefit. Whilst we previously demonstrated that anti-sera generated in mice following immunisation with scFv can recognise autologous Ig (using low-grade non-Hodgkin lymphoma sequences as the source of idiotypic protein-unpublished observations), our data here suggest there may be a difference between recognition of whole Ig and scFv, since scFv is seen but not paraprotein. This may be due to differences in protein folding, with novel, immunogenetic B-cell epitopes revealed in scFv that are not visible on the human paraprotein.

The development of an anti-Id cellular immune response is believed to be of greatest importance for the effective treatment of MM; the lysis of autologous myeloma plasma cells by Id-specific T cells [14] and a correlation between vaccine-induced Id-specific T cells and a reduction in circulating myeloma cells in patients [37] have been demonstrated. We report vaccine-induced anti-Id T-cell responses for three patients, detectable with either scFv (*n* = 1) or paraprotein (*n* = 2). Pre-clinically, using the murine MOPC-315 plasmacytoma model, Bogen et al. [38] demonstrated that vaccine-induced T-cell responses to an Id of the myeloma protein M315 were heavily influenced by the quaternary structure of the stimulating protein in a proliferation assay, with a 100-fold to 1000-fold higher molar concentration of Fab or whole IgA needed to induce equivalent responses to Fv, suggesting that whole Ig is poorly processed in vitro, and possible in vivo, which may explain the relatively low frequency of anti-Id responses observed. These data may also be relevant for understanding the differential Ab responses to scFv and paraprotein noted earlier. Naturally occurring Id-specific CD4^+^ T cells have been detected by proliferation and/or ELISPOT assay in the blood of previously untreated MM patients with stage I or II disease [18, 39]. Hansson et al. [18] report that Id-specific T cells were associated with patients with serum paraprotein of below 50 g/L, indicating that idiotypic vaccination may be more relevant for patients with a low tumour burden. Patients with pre-existing anti-Id T cells may respond better to vaccination as it may be easier to boost rather than induce de novo immunity against weak self-antigens. Indeed, vaccine-induced Id-specific T-cell responses are more frequently reported in patients that exhibit a pre-existing anti-Id T-cell pool [18, 40]. In our study, we did not observed any pre-existing Id-specific cellular immune responses although all patients had serum paraprotein of <50 g/L, suggesting that vaccination stimulates a new pool of T cells against Id. The hurdle for priming an immune response to Id is probably much greater than that for a recall response; this likely contributes to the difference in magnitudes between anti-FrC and anti-Id responses. However, it is important to remember that in our study patients had previously received ASCT/HDT and were 6.4–21.4 months post-therapy.

Clinical trials employing idiotypic vaccination have shown mixed responses in follicular lymphoma and MM thus far [9–12]. Recent trials of idiotypic vaccination in MM have used Id protein coupled to immunogenic carriers, such as key hole limpet haemocyanin and filamentous phage, in combination with adjuvant cytokines (GM-CSF and IL-12) and Id-pulsed dendritic cells amongst others, and have shown that Id-specific T-cell responses can be generated in approximately 50 % of patients [40, 41]. Conversely, clinical responses were observed in only 12 % of patients, demonstrating that Id-specific responses do not always give rise to clinical benefit. We demonstrate that Id-specific T cells are associated with a reduction in serum paraprotein in one patient. Although we cannot definitively conclude that the presence of Id-specific T cells is causative, this is highly implied in the absence of further clinical intervention. Increases in Id-specific T cells can correlate with reduced numbers of circulating myeloma cells, even without a reduction in serum paraprotein or evidence for a survival benefit [37]. Conversely, clinical responses have been seen without the induction of measurable immune responses [40]. Overall in our study, the stabilisation or decrease in paraprotein in MM patients following idiotypic vaccination is encouraging, but since patients had previously received ASCT/HDT it is difficult to dissect the effects of the vaccine from that of a delayed response to treatment and, therefore, they must be interpreted with caution.

Improved immunotherapeutics for myeloma are clearly needed. One option would be to use a generic target for DNA fusion vaccination, such as CS1, CD38 or CD138 [42], to avoid the need for bespoke vaccine production. A second approach could be to increase vaccine efficiency by delivery of DNA vaccine with electroporation, which has previously shown to amplify immune responses induced by therapeutic cancer vaccines [43]. Recent data indicate that deep and prolonged clinical responses can be achieved using the immunomodulatory drug lenalidomide as part of induction pre-ASCT and maintenance therapy [44]. Given that lenalidomide has also been demonstrated to augment responses to pneumococcal vaccination through its immunomodulatory effects, a combination approach using Id vaccination alongside immunomodulatory agents is attractive [45]. Our data will be useful for the design of clinical trials powered to assess effect of idiotypic vaccination on improving clinical endpoints, with the hope of developing a therapeutic vaccination strategy to delay disease progression.

## Electronic supplementary material

Supplementary material 1 (PDF 691 kb)

## Abbreviations

Ab: Antibody
AE: Adverse event
ASCT: Autologous stem cell transplantation
BM: Bone marrow
CR: Complete response
FrC: Fragment C of tetanus toxin
HDT: High-dose chemotherapy
Id: Idiotype
Ig: Immunoglobulin
MIATA: Minimal information about T-cell assays
MM: Multiple myeloma
OS: Overall survival
PBMC: Peripheral blood mononuclear cells
PR: Partial response
scFv: Single chain variable fragment
SD: Stable disease
SFC: Spot-forming cells
TTP: Time to progression
*V* genes: Variable region genes
*V*_*H*_: Heavy chain variable region gene
*V*_*L*_: Light chain variable region gene
