# Supplementary material for: Idiotypic DNA vaccination for the treatment of multiple myeloma: safety and immunogenicity in a phase I clinical study
Source: Cancer Immunol Immunother. 2015 May 16;64(8):1021–32. doi: 10.1007/s00262-015-1703-7 (PMC4506484; doi:10.1007/s00262-015-1703-7)
Supplement: Supplementary file 1 — Supplementary material 1 (PDF 691 kb) [file 262_2015_1703_MOESM1_ESM.pdf]

Cancer Immunology, Immunotherapy (submitted in 2015) – Katy McCann *et al.*

Supplementary Figures

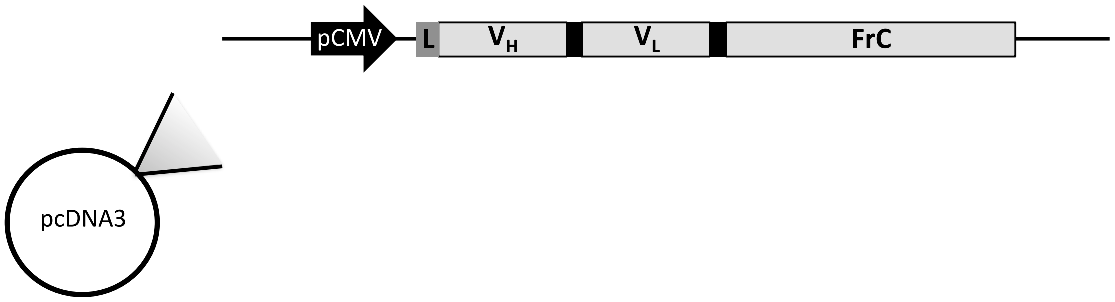

Supplementary Fig. 1 DNA fusion vaccine design Tumour-derived V_H and V_L gene sequences were assembled as scFv, linked via a 45-nucleotide linker; the human *VH1-46* leader featured upstream of V_H [7, 20]. The *FrC* sequence of tetanus toxin was linked to scFv at the C-terminus via a 12-nucleotide linker. *scFV-FrC* was cloned into pcDNA3 vector containing an upstream human CMV-derived promoter.

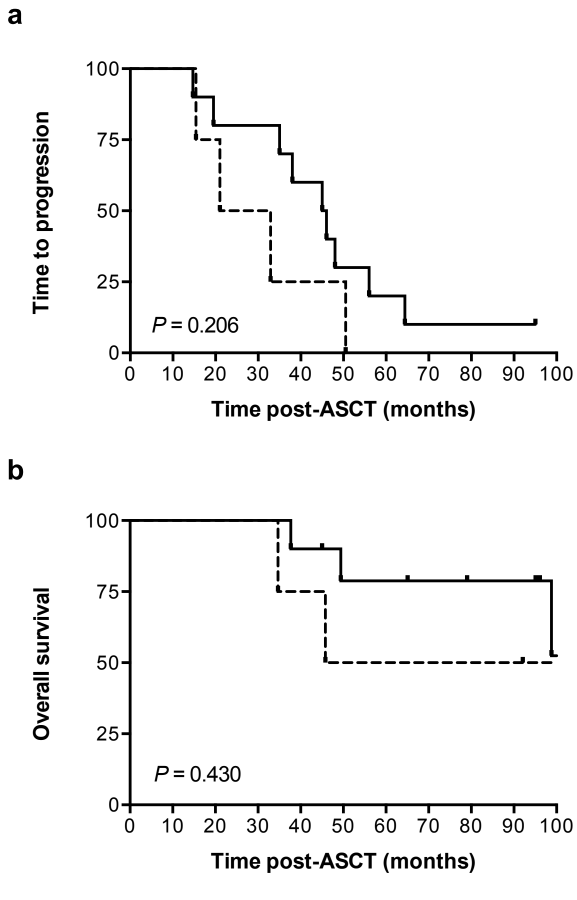

Supplementary Fig. 2 Clinical outcome TTP and OS were recorded for each patient on study to event or censor date; data was frozen in Sept. 2013 for analysis. a) A Kaplan-Meier plot of TTP, defined as the time from the date of ASCT to progression of paraprotein, according to International Myeloma Working Group criteria [30]. b) A Kaplan-Meier plot of OS, defined as the time from the date of ASCT to death. The small *vertical tick* marks show censored times. The *dashed line* represents Id-responders (n=4) and the *solid line* Id non-responders (n=10).

Supplementary MIATA Information

MODULE 1: MINIMAL INFORMATION ON THE SAMPLE

IA: DONOR

Patient demographics are reported in Table 1.

1B: SOURCE

At each study visit and for each of the vaccinated patients blood was collected in Lithium/Heparin tubes. The time between drawing and processing blood was <4hrs. PBMCs were purified by density gradient centrifugation using Lymphoprep™ (Axis-Shield PoC AS, Oslo, Norway), counted and viability assessed prior to freezing using manual haemocytometer and 0.4% trypan blue stain (Sigma-Aldrich Company Ltd., Gillingham, UK).

1C: CRYOPORESERVATION AND STORAGE

PBMCs were frozen at a concentration of 5-10 x10^6 per vial in 50% human AB serum (Sigma-Aldrich Company Ltd., batch tested) 40% complete RPMI 1640 (RPMI plus sodium pyruvate, penicillin, streptomycin and L-glutamine; see section 2B) and 10% DMSO. Freezing additives were added at room temperature. Freezing followed a step-wise drop in temperature to –80°C using a Nalgene® Mr Frosty® (Thermo Fisher Scientific, Roskilde, Denmark). After ~24 hours, PBMCs were transferred to Liquid N_2 where they were stored until the end of follow-up (week 52) and prior to assay. PBMCs were stored in Liquid N_2 for a mean period of 28 months (range 12-50 months) prior to thawing and assay -duration was calculated based on baseline (week 0) PBMCs.

1D: QUALITY OF CELL MATERIAL

Mean recovery of PBMCs after thawing was 70% (range 25-144%), expressed as a percentage of initial PBMC input before freezing. Mean viability of PBMCs after thawing was 91% (range 54-100%).

MODULE 2: MINIMAL INFORMATION ON THE ASSAY

2A: CELL COUNTING

Manual cell counting/viability assessment used a haemocytometer and 0.4% trypan blue stain. See also section 1D.

2B: MEDIUM/SERUM

Ex vivo ELISPOT used RPMI 1640 (PAA Laboratories GmbH, Pasching, Austria) containing 1mM sodium pyruvate, 100U/mL Penicillin – 0.1mg/mL streptomycin and 2mM L-glutamine (all Sigma-Aldrich Company Ltd.) plus 10% Human AB Serum (Sigma-Aldrich Company Ltd., batch tested).

2C: THE ASSAY

Ex vivo ELISPOT*:* All assays were performed using cryopreserved PBMCs. Clear Multiscreen 96-well ELISPOT plates (MAIPS4510; Millipore (UK) Ltd., Watford, UK) were pre-coated with 15 μg/ml anti-human IFN-γ antibody* (mAb 1-D1K; MABTECH AB, Nacka Strand, Sweden) overnight at 4°C. PBMCs (4x10^5/well) were incubated in triplicate with medium only, recombinant FrC protein (20μg/mL), recombinant scFv protein (100μg/mL), irrelevant scFv control protein (100μg/mL), patient-derived Ig paraprotein (100μg/mL), irrelevant isotype matched Ig paraprotein (100μg/mL) and phytohemagglutinin (PHA; 5μg/mL) (Sigma Aldrich Company Ltd.) for 40 hr at 37ºC in 5% CO_2. IFN-γ-secreting memory T cells specific for the antigen were detected as spots using 1 μg/ml biotinylated IFN-γ antibody* (mAb 7B61 biotin, MABTECH AB) followed by 1 μg/ml streptavidin alkaline phosphatase (MABTECH AB) followed by BCIP/NBT detection kit (Zymed GmbH, Rennbahnweg, Vienna, Austria).

*IL-2 and IL-13 ELISPOT assays used the Human IL-2 ELISpot (ALP) kit (mAb MT2A91/2C95 and mAb MT8G10) and Human IL-13 ELISpot (ALP) kit (mAb IL13-I and mAb IL13-3), respectively (MABTECH AB).

N.B. Recombinant FrC and scFv proteins (tagged with kappa chain constant region) were generated in-house using the mammalian Freestyle 293 expression system (Invitrogen Ltd.), according to the manufacturer's instructions. Purification was using polyclonal sheep anti-human free kappa linked to Sepharose 4B beads.

2D: CONTROLS

A negative control of media only and a positive control of PHA (5μg/mL) (Sigma Aldrich Company Ltd.) were used. Idiotype controls used an irrelevant scFv control protein (100μg/mL) and an irrelevant isotype matched Ig paraprotein (100μg/mL).

MODULE 3: DATA ACQUISITION

3A: EQUIPMENT AND SOFTWARE

Spot forming cells (SFC) were counted using the AID ELISpot reader System ELR04 and AID ELISpot software version 4.0 (AutoImmun Diagnostika GmbH, Strassberg, Germany). Universal spot count settings, including spot intensity, size and gradient, were pre-defined and used as standard for all patients on study; T-cell responses of buffy coat donors to the common viral antigens FLU, EBV and CMV were used to define count settings.

3B: ACQUISITION STRATEGY

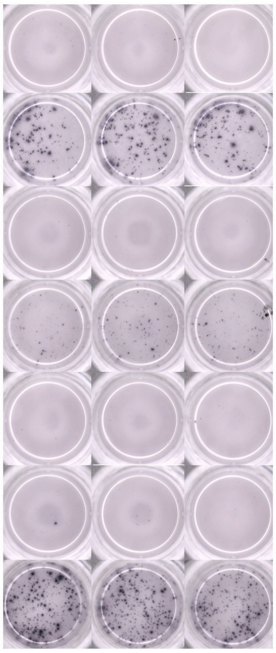

Example raw data: representative wells taken from *ex vivo* ELISPOT assay for MM05

MODULE 4: THE INTERPREATION OF RESULTS

4A: RAW DATA

Plates were read and data calculated as follows:

- SFC/well recalculated to SFC/million
- Mean of medium only control triplicate subtracted from antigen specific wells
- Mean and SDEV calculated for each triplicate for antigen-specific responses

Data exclusion applied on a maximum of three wells per plate e.g. an outlier could be removed from a triplicate.

Below is a table of IFN-γ SFC/million from all patients at all time-points following stimulation with media only or antigen (Id or FrC).

	All samples			Baseline			Weeks 2-52		
	**Median**	**Mean**	**Range**	**Median**	**Mean**	**Range**	**Median**	**Mean**	**Range**
**Background** (medium only)	13	24	0-206	6	20	1-103	13	24	0-206
**Id-specific response**	0	11	0-385	0	3	0-38	0	12	0-385
**FrC-specific response**	49	76	0-309	27	62	0-270	50	77	0-309

Raw data may be provided upon request.

4B: RESPONSE DETERMINATION, STATISTICAL TESTS AND EMPIRICAL RULES

Idiotype-specific responses were obtained by deducting SFC for matched, irrelevant control proteins prior to analyses.

A positive response to an antigen at any given time-point was defined as mean SFC minus mean SFC of pre-vaccination baseline (week 0) =

- greater than 20 SFC/million
- greater than 2 SDEV above medium only wells
- Statistically significant ($p<0.05$); Statistical analyses were performed with the GraphPad Prism software program using a Student's t-test

The definition criteria for a positive response was pre-defined before the study commenced:

- A patient showing a negative response has no time-points that meet the above criteria
- A positive [+] responder has a single time-point that meets the above criteria
- A strong positive [++] responder has more than one time-point that meets the above criteria

MODULE 5: THE LABORATORY ENVIRONMENT
**5A: GENERAL LABORATORY OPERATION**
This study was conducted in a laboratory that operates under GCLP principles.
**5B: LABORATORY PROCEDURE STANDARDISATION**
This study was performed using standard operating procedures.
**5C: STATUS OF ASSAY QUALIFICATION AND VALIDATION**
This study was performed using optimised and validated assays.
